# Supplementary material for: Cellular barcoding of protozoan pathogens reveals the within-host population dynamics of Toxoplasma gondii host colonization
Source: Cell Rep Methods. 2022 Aug 22;2(8):100274. doi: 10.1016/j.crmeth.2022.100274 (PMC9421581; doi:10.1016/j.crmeth.2022.100274)
Supplement: Document S1. Figures S1 and S2 and Table S1 [file mmc1.pdf]

Cell Reports Methods, Volume *2*

Supplemental information

Cellular barcoding of protozoan pathogens reveals the within-host population dynamics of *Toxoplasma gondii* host colonization

Ceire J. Wincott, Gayathri Sritharan, Henry J. Benns, Dana May, Carla Gilabert-Carbajo, Monique Bunyan, Aisling R. Fairweather, Eduardo Alves, Ivan Andrew, Laurence Game, Eva-Maria Frickel, Calvin Tiengwe, Sarah E. Ewald, and Matthew A. Child

Infection timepoint:	48 hours			28 days													
Mouse #	1	2	3	1	2	3	4	5	6	7	8	9	10	11	12	13	14
Barcode #	-	79	-	-	-	8, 60, 71	-	8, 36	-	-	79	-	60, 71, 79	-	-	-	22
Total read #	$14x10^6$	$0.59x10^6$	$2.3x10^6$	$8.3x10^6$	$10x10^6$	$0.62x10^6$	$6.3x10^6$	$0.46x10^6$	$8.0x10^6$	$3.3x10^6$	$1.8x10^6$	$4.1x10^6$	$0.73x10^6$	$5.0x10^6$	$1.7x10^6$	$4.2x10^6$	$0.92x10^6$

Table S1: Details of barcode extinctions occurring during the different phases of infection. Barcode numbers and the mouse in which the extinction was observed are noted as shown in Figure 2C-D. Extinctions are defined by an absence of the barcode sequence within the processed NGS read data. Related to Figure 3.

Table S2: Sequences of barcoding oligo nucleotides.
Table S3: Raw data from all NGS experiments.

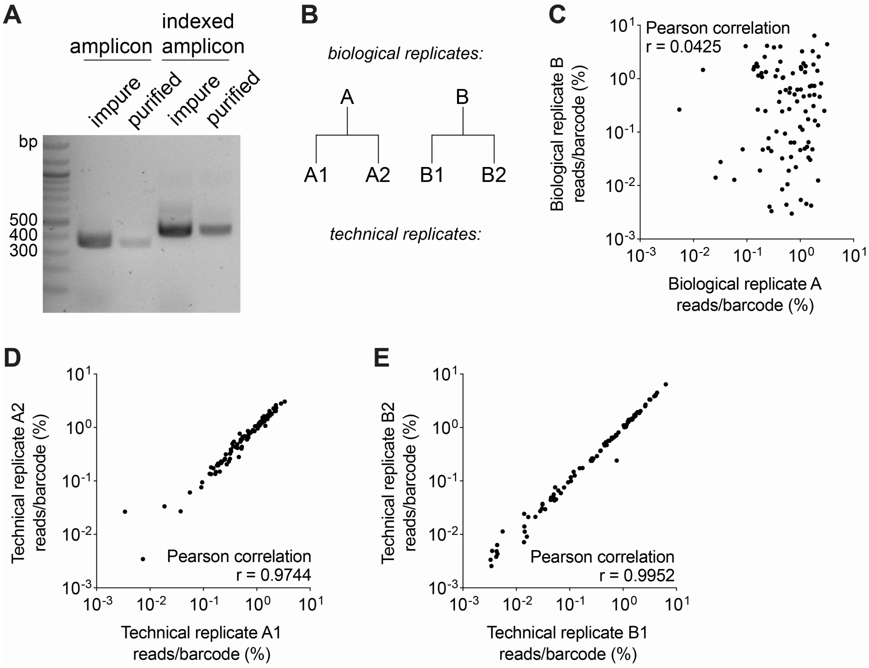

Figure S1: A robust pipeline for analysis of barcoded *T. gondii* libraries by NGS. Related to Figures 1 and 2. **A)** A single ~300 bp region of the UPRT locus was amplified from genomic DNA and purified (amplicon, lanes 1 and 2). The purified amplicon was then indexed and re-purified prior to quantification and sizing (indexed amplicon, lanes 3 and 4). **B**) Strategy to isolate the primary source of variation to the multiplexed transfection barcoding strategy or the NGS pipeline. **C-E**) Scatter plots show the percent representation of individual barcodes within library pools for biological replicate transfections, (**C**, PCC r = 0.0425, n = 96, P (two-tailed) = 0.6807), or technical replicates of amplicon indexing (**D**, PCC r = 0.9744, n = 96, P (two-tailed) = <0.0001) and (**E**, PCC r = 0.9952, n = 96, P (two-tailed) = <0.0001). PCC values represent comparison between samples indicated on each x and y axis.

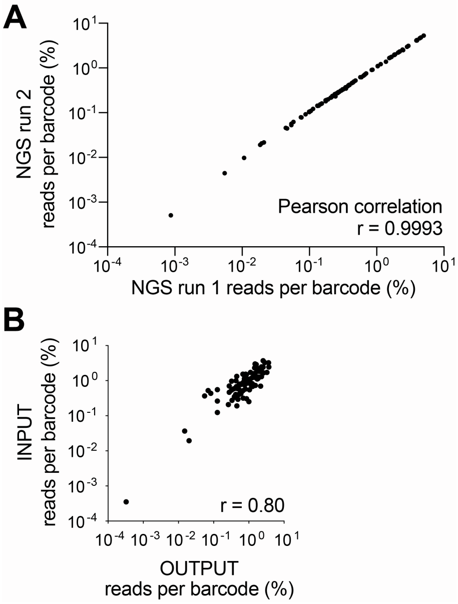

Figure S2: Analysis of NGS pipeline reproducibility and one-pot barcoded strain library long-term stability. Related to Figure 2. **A)** Scatter plot of relative percentage frequency of barcodes within a single genomic sample processed on independent NGS runs. PCC r = 0.99, n = 96, P (two-tailed) = <0.0001. **B)** Scatter plot comparing 96 individual barcode frequencies within the INPUT one-pot library population and the OUTPUT sample harvested after 28 days (~19 x 36-hour lytic growth cycle), PCC r = 0.80, n = 96, P (two-tailed) = <0.0001).
